# The unfulfilled promises of scorpion insectotoxins

**DOI:** 10.1186/s40409-015-0019-6

**Published:** 2015-06-17

**Authors:** Ernesto Ortiz, Lourival D Possani

**Affiliations:** Departamento de Medicina Molecular y Bioprocesos, Instituto de Biotecnología, Universidad Nacional Autonóma de México, Avenida Universidad 2001, Cuernavaca, 62210 Mexico

**Keywords:** Bioinsecticides, Insectotoxins, Scorpion venom

## Abstract

Since the description and biochemical characterization of the first insect-specific neurotoxins from scorpion venoms, almost all contributions have highlighted their potential application as leads for the development of potent bioinsecticides. Their practical use, however, has been hindered by different factors, some of which are intrinsically related to the toxins and other external determinants. Recent developments in the understanding of the action mechanisms of the scorpion insectotoxins and their bioactive surfaces, coupled with the exploration of novel bioinsecticide delivery systems have renewed the expectations that the scorpion insectotoxins could find their way into commercial applications in agriculture, as part of integrated pest control strategies. Herein, we review the current arsenal of available scorpion neurotoxins with a degree of specificity for insects, the progress made with alternative delivery methods, and the drawbacks that still preclude their practical use.

## Introduction

Insects are the most diverse class of animals living on Earth, with more than one million described species. They are highly adaptable and successful, easily outnumbering any other animal category [1].

Documents distributed by the World Health Organization report many cases of insects that are disease-transmitting vectors and represent a great menace to human populations [2]. Mosquitoes are the most relevant, since they can transmit malaria, dengue and yellow fever. Together these three illnesses account for hundreds of millions of cases and several million deaths every year. Mosquitoes also spread lymphatic filariasis and Japanese encephalitis. Other parasites are carried by different insects. The tsetse fly transmits the African trypanosomiasis or sleeping sickness that causes around 9 thousand deaths per year. The American trypanosomiasis, more commonly known as Chagas’ disease, is spread mostly by blood-sucking insects known as Triatominae or kissing bugs. At least 16 million people in Latin America are infected with Chagas’ disease, and more than 10 thousand patients die of Chagas’ every year. Leishmaniasis is spread by the bite of certain types of sandflies. It causes the death of between 20 and 50 thousand persons every year. Onchocerciasis, or river blindness, is carried by blackflies. About 17 to 25 million people are nowadays infected with river blindness, mostly in sub-Saharan Africa, with approximately 0.8 million having some amount of loss of vision. Plague, the deadly infectious disease propagated by fleas that has decimated the human population through history, is still endemic in some parts of the world [2]. Other insects that constitute disease agents for humans include lice and bed bugs.

The direct damage caused by insect pests to agriculture has been estimated by various authors to be responsible for the loss of over 15 % of the global food production [3–5]. This number does not consider the secondary losses caused by plant diseases transmitted by insects. The threat of insect damage to agriculture is expected to increase as the planet warms and high-yielding varieties expand into less suitable regions, replacing well-adapted and more resistant local varieties [5]. According to the most recent United Nations 2012 Revision of the World Population Prospects [6], the world population will reach 9 billion around 2040 and will continue to grow until it stabilizes at just above 10 billion persons. In order to feed that population, the crop yields must increase by at least 40 %. This cannot be achieved without a rational and integrated pest/crop management, including crop protection through biological and chemical measures [4].

The use of synthetic insecticides dates back to the introduction of dichlorodiphenyltrichloroethane (DDT) at the end of World War II. Different generations of synthetic insecticides have helped mankind to control the burden of insect pests, although not without consequences for the environment [7]. Their indiscriminate use and the high frequency with which insecticides have been applied have led to the emergence of insect strains resistant to their active principles [8, 9]. There is no other way to prevent or at least delay the emergence of resistance other than the alternated use of substances with different mechanisms of action, combined with integrated pest control strategies, including the protection of beneficial organisms and the pest’s natural antagonists. It is in this context that scorpion venom insect-specific toxins (insectotoxins), with particular modes of action, have become attractive candidates for the development of novel insecticides.

Scorpions constitute a very well adapted order of predatory animals. They have inhabited the planet for well over 400 million years, being among the first complex animals to make the transition from sea to land [10]. They are so successful that their morphology has changed little over this long timespan. Meanwhile, they have diversified to comprise more than 1700 species that have been described to date [11]. The key to their success is the production of potent and complex venoms that they use primarily to kill or paralyze their prey and to deter possible competitors and predators. Insects constitute an important food source for most scorpion species, and therefore, potent peptidic insectotoxins have been isolated from the venoms of different scorpion species.

These toxins are valuable as leads for the development of insecticides. Their practical application, however, has been hindered by problems mainly associated with their natural mechanism of delivery. Scorpions inject venom into their prey with a stinger, so the toxins did not naturally evolve to be resistant to the harsh conditions of the insect’s digestive system. Therefore, alternatives to feeding, or other variants with enhanced cuticle or gut mucosa absorption have to be devised. The other problem that has to be circumvented is their potential broad range of targets, which may include other beneficial insects or even mammals. It is highly relevant to accurately determine the principles that sustain their specificity, which includes the determination of their interacting surfaces with the target receptors. Recent advances have been made in both areas, with novel delivery methods and studies of the structural determinants of highly selective insectotoxins reported.

## Review

### Scorpion venom peptidic insectotoxins

Scorpion neurotoxins (ScTxs) are classified according to their pharmacological target into long-and short-chain toxins. Long-chain toxins (61 to 76 amino acids) modify the gating mechanism of voltage-gated sodium (Na_v_) channels [12]. Short-chain toxins (28 to 46 amino acids) primarily block potassium channels [12]. Based on the physiological effect that the long-chain toxins elicit on Na_v_ channels, they are further classified as alpha (α-NaScTxs) or beta (β-NaScTxs). The α-NaScTxs target the receptor site 3 of Na_v_ channels and inhibit the channel’s rapid inactivation process, thereby prolonging the action potential [13]. The β-NaScTxs bind to receptor site 4 and shift the channel activation to more negative potentials [14].

Only a few α-NaScTxs exhibit high activity in insects. Examples include LqhαIT from *Leiurus quinquestriatus hebraeus* [15] (currently denominated *L. hebraeus* [16]), Lqq3 from *L. q. quinquestriatus* [17] (currently named *L. quinquestriatus* [16]), BotIT1 from *Buthus occitanus tunetanus* [18] (currently denominated *B. tunetanus* [19]) and BjαIT from *Buthotus judaicus* (now known as *Hottentotta judaicus*) [20]. These toxins are highly active on insects but are weak on mice (as tested by intracerebroventricular injection), and bind with high affinity to insect neuronal preparations but weakly to rat brain synaptosomes [21]. These properties are in sharp contrast with the effects produced by the majority of the reported “classical” α-NaScTxs, which are very potent on mammalian Na_v_ channels, bind with high affinity to rat brain synaptosomes, and show strong toxicity to mammals while presenting very weak toxicity when injected to insects.

Scorpion α-insectotoxins and the classical α-NaScTxs share the same cysteine-stabilized αß scaffold, while their three-dimensional structures are very similar in spite of their sequence diversity. Their pharmacological differences seem to be related to small structural differences in limited regions of the toxins and slight alterations in their surface topology [21, 22]. Small differences in the receptor site 3 in the homologous yet non-identical insect and mammalian Na_v_ channels might be selectively discriminated by the different α-NaScTxs. Unfortunately, detailed structural studies comparing receptor site 3 between insects and mammals are not available. Moreover, due to the flexibility displayed by protein-protein interactions, it is possible that rearrangements occur after toxin-to-channel binding, so these studies would have to be performed on the channel-toxin complexes, which adds a new level of complexity.

Finally, differences in receptor site 3 from Na_v_ channels in different insect species have also been revealed by the variations in binding affinity of α-insectotoxins to neuronal preparations from different insects [21]. This emphasizes the importance of performing the structural-functional studies individually, an effort that could lead to insecticides specific for different insect orders, a very desirable outcome. The recent publication of the crystal structures of bacterial Na_v_ channels demonstrates that the latest improvements in technology are bringing closer the long awaited goal of having a structure for their larger eukaryotic equivalents [23–25]. Only then, fine structural-functional studies of scorpion insectotoxins’ interactions with their receptors will allow the rational design of potent specific insecticides derived from them.

The insect-active α-NaScTxs highlight the challenges of designing highly selective insecticides from scorpion toxins. Although they are significantly less toxic than classical (mammal-active) α-NaScTxs when injected intracerebroventricularly, the two classes are nevertheless similarly toxic to mice when injected subcutaneously, an undesirable characteristic that must be addressed [26].

There are two classes of β-NaScTxs that specifically affect insect Na_v_ channels and can be of interest as leads for the development of insecticides. The anti-insect excitatory β-NaScTxs are highly specific for insects. They provoke a frequent premature activation of Na_v_ channels at more negative membrane potentials in motor neurons causing excessive muscle contraction, which results in spastic paralysis [27, 28]. These toxins display no apparent activity when intracerebroventricularly or subcutaneously injected into mice, even at high concentrations [29]. Their selectivity has been associated with a structural element that sets them apart from the other long-chain toxins: an extra short α-helix at the C-terminus anchored to the N-terminal module by a shifted disulfide bridge [30]. Their high affinity and the total discrimination of insects *versus* mammal Na_v_ channels makes them excellent leads for the design of potent specific insecticides [31]. Examples include AaHIT from *Androctonus australis hector* [32], LqqIT1 from *L. quinquestriatus* [33], Lqh-xtrIT from *L. hebraeus* [34] and Bj-xtrIT from the species now known as *H. judaicus* [30].

The second class corresponds to the anti-insect depressant β-NaScTxs. These toxins induce flaccid paralysis when injected into insects. When assayed *in vitro* via insect neuron preparations, they depolarize the axon membrane, block the evoked action potentials and modify the amplitude and kinetics of the sodium current. The physiological effects on insects are the result of Na_v_ channels slowly opening at more negative potentials and not inactivating normally [35]. Examples of anti-insect depressant β-NaScTxs include LqhIT2 from *L. hebraeus* [36], BjIT2 from *H. judaicus* [36], BotIT2 from *B. tunetanus* [37] and BaIT2 from *Buthacus arenicola* [38]. The depressant β-NaScTxs were traditionally considered to be insect-selective, since individual toxins were not only toxic only to insects but also bind insect Na_v_ channels with high affinity [36]. However, it was later demonstrated that these toxins also bind the rat skeletal muscle Na_v_ channels with high affinity. Moreover, when those channels are preconditioned with a long depolarizing prepulse, the toxins exert their habitual action, shifting the activation towards more negative potentials [39]. This means that in the context of the whole venom, the depressant β-NaScTxs may have a toxic impact on mammals. Again, as in the case of the α-NaScTxs, this “specificity” issue has to be addressed before these toxins can be considered as leads for insecticides.

It is remarkable that all the aforementioned insect-active NaScTxs were identified from scorpions belonging to the Buthidae family. This family includes among its members some of the scorpion species most lethal to humans. Interestingly, there is a reported insect-specific scorpion toxin from a non-buthid scorpion, namely phaiodotoxin (PhTx), which was isolated from the venom of the *Anuroctonus phaiodactylus* scorpion (now called *A. bajae* [40]), a member of the Chactidae family (this species has sometimes been misclassified as a member of the Iuridae family). Two other putative isoforms, labeled PhTx2 and PhTx3, were identified from cDNA when cloning PhTx. Phaiodotoxin is a distinct long-chain toxin that shares low sequence similarity with α-NaScTxs (30-49 % similarity) and β-NaScTxs (21-38 % similarity), and has a unique disulfide bridge, and thus has been suggested as defining a new class of long-chain toxins [41].

Phaiodotoxin induced flaccid paralysis when injected into crickets and proved to be lethal at a dose of 1 μg per animal (weighing approximately 100 mg). On the other hand, phaiodotoxin was not active on mice, even when relatively large amounts (100 μg per 20 g of mouse) of the toxin were injected intraperitoneally. It also showed no effect on sodium currents when tested in several mammalian cell lines. Coincidentally, at least in Baja California, Mexico, there are no reported cases of intoxication in humans after stings of the *A. bajae* scorpion, suggesting that phaiodotoxins are insect-specific. It is intriguing that phaiodotoxin, being similar in sequence to α-NaScTxs and to β-NaScTxs, combines their physiological actions: it activates the insect Na_v_ channels at more negative potentials (the effect of β-NaScTxs) and delays their inactivation (as α-NaScTxs do). For insect Na_v_ channels expressed in *Xenopus* oocytes, the window current is increased 225 % when 2 μM PhTx is added, with respect to the control without the toxin [41]. This should result in a powerful interference with the transmission of the action potentials and should lead to the death of the insects.

The notable specificity and potency of Phaiodotoxin might indicate that the search for insect-specific scorpion toxins that could serve as leads for the development of insecticides would have to be shifted to scorpion species that are not toxic to mammals, in order to minimize their potential adverse effects. Most of the more than 1700 scorpion species described thus far fall into this category. They represent an almost unexplored reservoir of toxins, some of which might display the desirable properties of selective and potent insecticides.

### Delivery methods

Neurotoxins are delivered as part of the whole scorpion venom by stings. They are rapidly spread through the circulatory system of the victim (hemolymph in insects) until they reach their molecular targets. They have not evolved to ensure high oral bioavailability. In this sense their practical applications face tough competition with other toxins, such as the δ-endotoxins from the *Bacillus thuringiensis* (*Bt*) bacteria that, on the contrary, depend on oral ingestion for delivery and require the alkaline conditions of the insect gut to be solubilized and proteolytically activated [42]. Scorpion neurotoxins are also unlikely to be rapidly absorbed through the target insect’s cuticle, and would be prone to degradation in the environment. Consequently, they are not expected to be effective as components of insecticidal sprays. Scorpion toxins need to be engineered for good oral bioavailability or alternative delivery systems have to be devised.

Oral delivery presents obvious advantages for crop protection since the insect-specific toxins may be present in, or sprayed on, plant tissues that are susceptible to damage. One mechanism of improving oral bioavailability is to fuse the toxin to a carrier protein able to translocate to the hemolymph after feeding. This strategy was successfully demonstrated with SFI1, a neurotoxin from the spider *Segestria florentina*, fused to the snowdrop lectin (*Galanthus nivalis* agglutinin, GNA). Whereas neither GNA nor SFI1 alone showed acute toxicity when fed to tomato moth (*Lacanobia oleracea*) larvae, the SFI1/GNA fusion was insecticidal and caused 100 % mortality to first instar larvae [43]. The same fusion protein was then fed to rice brown planthopper (*Nilaparvata lugens*) second- and third-instar nymphs, and to peach-potato aphid (*Myzus persicae*) neonate nimphs, with equal success [44].

Soon after, this system was tested with the scorpion short-chain toxin ButaIT from *Mesobuthus tamulus*. Although ButaIT has been claimed to be lepidopteran-specific [45], the fusion protein ButaIT/GNA was toxic when fed to lepidopteran larvae (*L. oleracea*) and also to the homopteran *N. lugens*, thus showing a wider range of insecticidal activity. The intact ButaIT/GNA was present in the hemolymph of insects fed a diet containing the fusion protein, showing that transport from the gut had occurred, although some proteolysis of the fusion protein was also observed [46]. In a more recent study comparing the insecticidal activities of the SFI1/GNA and ButaIT/GNA, it was shown that the fusion with the scorpion toxin was more effective than the one with the spider toxin. The ButaIT/GNA displayed low specificity, being active in lepidopteran, dipteran, coleopteran and dictyopteran pests, showing similar levels of activity across the different insect orders [47]. Yet another example of a neurotoxin considered “specific” turned out to have a broader spectrum of targets.

Research on delivery systems able to enhance protein translocation across the insect digestive system is just starting to gain momentum. The use of lipophilic polyethylene glycol (PEG) polymers, protease inhibitors, the development of fusion proteins with lectins (as shown with GNA), and the development of amphiphilic peptide analogs are among the approaches already successfully tested [48]. All the above-mentioned reports on toxins fused to GNA provide evidence that the development of fusion protein technology for the generation of new anti-insect moieties holds significant promise. It is adaptable to the generation of genetically modified plants, with all of their advantages: the long-term lower cost, the constant availability of the insecticide, the protection of the insectotoxins from environmental degradation and the limited collateral damage to non-pest species. Yet it is also feasible to apply the novel bioinsecticides via the traditional spraying methods, a very important feature in the face of widespread public reticence to the use of genetically modified organisms. There is still one unavoidable step before any of these fusion proteins can find their way to field testing: their oral toxicity to mammals, including humans has not been assayed. No toxicity towards higher animals is expected for two reasons. First, the toxins used are insect-specific. Second, it is known that the mammalian gut epithelium has very few binding sites for GNA, and therefore it is unlikely to be transported to the circulatory system [48]. The experiments to verify the innocuousness of the recombinant fusion proteins remain to be accomplished.

A strategy for insect control that avoids the introduction of foreign proteins into the food chain is the use of natural entomopathogenic organisms: viruses, bacteria, nematodes and fungi. The obvious choice is the engineering of baculoviruses, since they are arthropod-specific and do not infect vertebrates or plants [49]. Particular wild-type baculovirus strains have very restricted host ranges and can usually infect just a few insect species. They have already found applications in crop protection, although with limited success, due to the long time required for the infected insect to stop feeding on the crops. To accelerate the effects of the infection, recombinant baculoviruses have been engineered to express scorpion (among others) insect-specific neurotoxins. The *A. australis hector* excitatory β-toxin AaHIT was the first scorpion insectotoxin expressed in baculoviruses with clear biological activity [50–52]. It accelerated the velocity at which the control wild-type baculoviruses kill by 30-40 %, but most importantly, the infected larvae were paralyzed and stopped feeding very early, reducing the consumed leaf area by more than 60 % as compared to the wild-type infected larvae [53, 54]. Recombinant baculoviruses have been engineered to express the excitatory β-toxin LqhIT1 or the depressant β-toxin LqhIT2, or both [55, 56]. The viruses expressing the individual toxins reduced their time to kill by 24 % and 32 %, but the viruses that expressed both toxins further reduced the effective time until paralysis or death by 18-22 %, showing a synergistic effect of the toxins.

Notwithstanding the many advantages of the baculovirus system, and even when the expression of exogenous genes in recombinant baculoviruses has greatly improved the speed of insect incapacitation, the commercial application of this technology has stalled. Due to the widespread public aversion to the use of genetically modified organisms, the industry made a critical decision to not complete the registration process of recombinant baculoviruses for insect pest control [57].

Other delivery strategies of scorpion insectotoxins involving genetically modified organisms have been explored. The experimental direct expression of toxins in plants has already been undertaken with surprising, yet encouraging results. Transgenic cotton plants expressing the AaHIT toxin were shown to be more resistant to the damage by the larvae of the cotton bollworm (*Heliothis armigera*) than non-transformed plants [58]. LqhIT2 was expressed in recombinant rice under the control of the highly tissue-specific RuBisCO small subunit (rbcS) promoter. The expression was limited to the leaves, stems and roots, whereas no toxin was detected in mature seeds. The recombinant plants were much less prone to be attacked by the rice leaf roller (*Cnaphalocrocis medinalis*) larvae. In controlled experiments, the damage to the rice plants was reduced by up to 44 % as compared to the control plants, and in field experiments the damage to tiller and leaf was reduced by up to 40 % and 27 %, respectively [59]. These results are somewhat intriguing, since they seem to contradict the previous experience with direct toxin feeding, including the controls for the GNA fusions. The possibility of successfully protecting crops by the direct expression of scorpion insectotoxins in recombinant plants deserves further attention.

The genetic modification of enthomopathogenic fungi to express scorpion insect-specific neurotoxins has also been explored. The expression of LqhIT2 or BjαIT was shown to increase the virulence of *Metarhizium acridum* towards *Locusta migratoria manilensis*. In both cases, the transgenic fungal strains grew significantly faster in the insect’s hemolymph than the wild-type strain. For the LqhIT2-expressing strain, the median lethal times were reduced by 28 % and 30 % after topical inoculation and injection, respectively [60]. For the BjαIT-expressing strain, the median lethal times were reduced by roughly 30 % under both inoculation methods [61]. In spite of the compelling results obtained via modified *M. acridum* strains, the common issues associated with the approval of the release of genetically modified organisms are expected to be difficult to overcome by this strategy.

## Conclusions

The practical application of scorpion peptidic insectotoxins as insecticides is still far from a reality. The initial expectations surrounding the discovery and characterization of insect-specific scorpion neurotoxins have yet to be fulfilled. Some of the problems that hinder their commercial use are surely attributable to the toxins: their insect-specificity has yet to be proven without a reasonable, their innocuousness to non-pest organisms, including mammals, remains to be assayed, and their stability and oral bioavailability must be improved. However, there are also other barriers not related to the toxins. The resistance to accepting the use of genetically modified organisms by the general public, due to the real or perceived threats associated with this technology, has hindered the commercial use of mature, effective and proven strategies, such as the toxin-enhanced baculoviruses. It is still possible that these highly effective bioinsecticides will find their way to practical applications in agriculture, as public awareness and acceptance of genetically modified organisms increase.
